# Aggression and Adjustment Among Chinese Adolescents: The Role of Classroom Cultural Norms

**DOI:** 10.1007/s10802-025-01336-8

**Published:** 2025-06-12

**Authors:** Long Hei, Xinyin Chen, Junsheng Liu, Dan Li, Shihong Liu, Siman Zhao

**Affiliations:** 1 Human Development-Quantitative Methods Division, Graduate School of Education, University of Pennsylvania, 3700 Walnut St., Philadelphia, PA 19104-6216 USA; 2 School of Psychology and Cognitive Science, East China Normal University, Shanghai, China; 3 School of Psychology, Shanghai Normal University, Shanghai, China; 4 Department of Psychology, University of Dayton, Dayton, OH USA

**Keywords:** Aggression, Cultural orientations, Classroom norms, Adolescents’ development

## Abstract

Classroom environment may play a significant role in shaping adolescent development. This one-year longitudinal study investigated the moderating effects of classroom cultural norms on the relations between aggression and adjustment among Chinese adolescents. Participants included 2,671 students (47.7% boys) in middle schools, initially in 7th grade (*M* age = 12.91 years), in China. Data on self- and group-orientations, aggression, and adjustment variables were obtained from multiple sources including self-reports, peer nominations, teacher ratings, and school records. Classroom group-oriented norm significantly moderated the relations between aggression and later adjustment. More specifically, aggression was negatively associated with academic and social competence in classrooms with higher scores on group-oriented norm. Aggression was also positively associated with distinguished studentship and negatively associated with loneliness in classrooms with lower scores on group-oriented norm. The results suggested that adolescents who were more aggressive performed worse in classrooms with a higher group-oriented norm and better in classrooms with a lower group-oriented norm. The study indicates that the context of classroom may affect school and psychosocial adjustment of adolescents high on aggression.

## Introduction

Aggressive behavior during peer interactions emerges in the early years and becomes a salient and prevalent issue throughout childhood and adolescence (Dodge et al., 2006; Eisner & Malti, 2015). As it inflicts harm and causes conflicts during peer interactions, aggressive behavior is usually perceived and reacted to negatively by others. Adolescents who are aggressive are likely to be rejected by peers and considered immature and incompetent by adults and experience difficulties in school functioning (Landsford et al., 2010; Rubin et al., 2015). Research has consistently shown that adolescents with high aggression may develop a wide range of negative outcomes, such as externalizing problems, low educational achievement and occupational status, and poor quality of social relationships (e.g., Dodge et al., 2006; Eisner & Malti, 2015; Henriksen et al., 2021).

Whereas aggressive behavior is generally viewed negatively and related to adjustment problems, cross-cultural differences have been observed in the display and development of aggression (Bergeron & Schneider, 2005; Bergmüller, 2013; Ji et al., 2016). It has been found that aggression is more strictly prohibited in societies where group-oriented values are emphasized, largely because aggressive and disruptive behaviors may threaten the harmony and wellbeing of the group (Bergeron & Schneider, 2005; Chen & French, 2008). As a result, adolescents who display more aggressive behavior tend to experience more social difficulties and develop more maladaptive outcomes in group-oriented contexts. In the present study, we sought to explore how self-oriented and group-oriented cultural norms at the classroom level would moderate the associations between aggression and adolescents’ adjustment in China.

### Cultural Values in Chinese Context

China has traditionally been a collectivistic society where group-oriented values are highly emphasized. Children and adolescents are expected and socialized to learn social standards and behaviors that are conducive to harmonious group functioning while controlling their impulsive acts in social settings in Chinese society (Chen, 2023; Zhou et al., 2010). Findings of numerous studies have indicated that Chinese adolescents who display aggressive and disruptive behaviors have a variety of developmental problems, such as peer rejection, social incompetence, and academic difficulties (e.g., Chen et al., 2012a, 2012b; Yang et al., 2014). Moreover, due to negative social evaluation from peers, teachers, and parents, adolescents with a high level of aggression in China also tend to develop negative self-perceptions of their social competence and psychological problems, such as loneliness and depression (Chen et al., 2012a, 2012b; Teng et al., 2015).

China has undergone rapid development since the 1980s toward a market-centered economy. The requirements of the competitive social and economic environments and the influence of Western cultures as a result of increased globalization have led to a rise of self-oriented and individualistic values in the country (Cai et al., 2020; Greenfield, 2016). To help children and adolescents to develop skills that are needed to achieve success in the new environment, schools have started to revise the educational goals and practices to promote behavioral qualities such as initiative-taking and autonomy that have traditionally been neglected. Whereas self-orientation has been increasingly embraced, the traditional group-orientation remains to be valued and continues to play a significant role in shaping socialization and child development (Zhao et al., 2024). Empirical studies have shown that Chinese children and adolescents report high levels of both self-orientation and group-orientation (e.g., Liu et al., 2018). Moreover, self-orientation and group-orientation are positively associated (Liu et al., 2018; Tan et al., 2021), suggesting that Chinese children and adolescents may endorse self- and group-orientations at the same time. According to the pluralistic-constructivist perspective (Chen, 2024), the rising self-orientation and the traditional group-orientation may serve different functions in adolescents’ development. Whereas self-orientation may help adolescents achieve personal goals, group-orientation may help adolescents develop interpersonal connections and maintain group functioning.

The coexistence of self- and group-orientation is likely to be manifested in the classroom context. With classes varying on the levels of the orientations, the classroom context may serve to guide peer evaluation and regulation processes (Chen, 2012). Specifically, during social interactions, the group-level values provide a framework of reference for social judgements of individual behaviors. In the processes, peers display their attitudes of approval for behaviors that are regarded as appropriate and disapproval for behaviors that are regarded as inappropriate or deviant. The regulatory function of social interaction is reflected in children’s attempts to maintain or modify their behaviors according to peer evaluations. Children who fail to do so are likely to experience difficulties in social relationships and negative emotional reactions, such as frustration and distress, which may eventually lead to adjustment problems in school.

### Classroom Cultural Norms: Moderating Effects on Relations Between Aggression and Adjustment

According to Cialdini et al. (1991), the extent to which an attribute is approved/disapproved in the group represents a norm that is likely to guide social judgements and evaluations in interaction. Previous studies have shown that norm salience was an important factor in shaping adolescent development (Garandeau et al., 2022; Hu et al., 2023). For example, Hu et al. (2023) found that classroom sociable norm moderated the relations between adolescents’ unsociability and internalizing problems. More specifically, unsociable adolescents were more likely to develop a low level of self-esteem as well as high levels of depression and loneliness in classrooms where sociability was more preferred. Garandeau et al. (2022) found that bullying defending behaviors were more popular in classrooms where bullying was considered more unacceptable.

In the current study, we were interested in how classroom cultural norms would serve as a context as indicated in their moderating effects on the relations between aggression and adjustment in middle school students. Classroom cultural norms refer to the extent to which certain cultural values are endorsed or preferred by students in the classroom. In this study, we focused on the classroom group-oriented and self-oriented cultural values, which are highly relevant to adolescents’ social interaction and adjustment in the literature (e.g., Greenfield et al., 2006; Kagitcibasi & Ataca, 2005; Liu et al., 2018; Zhao et al., 2024). To our knowledge, this is the first study focusing on the interacting effects of aggression and classroom cultural norms. The years of middle school are a crucial period of development for adolescents as students engage in increasingly more extensive peer interactions and experience heighted pressure for social and academic achievement in school (e.g., Chen et al., 2023). At the same time, adolescents are becoming highly sensitive to social standards and social evaluations (Chen, 2012; Li et al., 2007). Research has also indicated that aggression is an important phenomenon in middle school as adolescents may engage in aggressive behavior as a strategy to achieve social status (Hawley, 2007; Moffitt, 1993; Ojanen & Nostrand, 2014). Thus, it would be interesting to investigate how aggression is associated with adjustment across domains in classrooms with different cultural norms. As different peer attitudes of self- and group-orientation are manifested through classroom cultural norms, we expected that the contextual effects of peer attitudes toward aggression in different classrooms would be manifested differently.

In the literature, an interactional model that is commonly used for contextual effects is the stress-buffering model (Cohen & Wills, 1985; Rutter, 2013). This model focuses on factors that buffer against or exacerbate the effect of the risk or adversity. For example, aggressive behavior was found to be more strictly prohibited in a group-oriented context and relatively more tolerated in a self-oriented context (Chen & French, 2008). In a classroom with a higher group-oriented norm or a lower self-oriented norm, aggressive behavior may be perceived as more unacceptable, leading to more negative evaluations by their peers. Thus, being in a classroom with a higher group-oriented norm or a lower self-oriented norm may increase the risk for children with high aggression, resulting in higher chances of developing adjustment problems. On the other hand, in a classroom with a higher self-oriented norm or a lower group-oriented norm, aggressive behavior may be considered less deviant, and children with high aggression may not receive the same level of social disapproval from peers. Thus, being in a classroom with a higher self-oriented norm or a lower group-oriented norm may reduce the risk of children with high aggression and thus protect them from developing problems. In this model, we would expect positive individual-level relations between aggression and later adjustment problems or negative individual-level relations between aggression and later positive outcomes in classrooms with a higher group-oriented norm or a lower self-oriented norm and weaker or nonsignificant individual-level relations in classrooms with a lower self-oriented norm or a higher group-oriented norm.

In addition to the stress-buffering model, the resource-potentiating model (Kupersmidt et al., 1995) focuses on factors that facilitate individuals’ development of adaptive processes. According to this model, in a classroom where self-orientation is preferred, aggressive behavior may be considered an expression of autonomy or a useful tool for achieving personal goals. A classroom with a higher self-oriented norm may potentiate the strengths of adolescents who use aggressive behavior as a strategy to obtain social status and other benefits in school (Hawley, 2007). In contrast, a classroom with a higher group-oriented norm or a lower self-oriented norm may hinder the development of positive adjustment of children high on aggression. Statistically, this model would be represented by significant individual-level relations (e.g., positive association between aggression and later positive adjustment or negative association between aggression and problems) in higher self-oriented classrooms or lower group-oriented classrooms and nonsignificant or weaker individual-level associations in higher group-oriented or lower self-oriented classrooms.

### The Present Study: An Overview

The main purpose of this one-year longitudinal study was to examine the role of classroom cultural norms in moderating the relations between aggression and later psychological, social, and school adjustment in Chinese adolescents. Research has shown that the classroom norms may play a significant role in adolescents’ development (Garandeau et al., 2022; Hu et al., 2023; Veenstra & Lodder, 2022). The variations in self-orientation and group-orientation among classrooms provided an excellent opportunity to examine the effects of cultural orientations at the group level on the associations between aggression and adjustment at the individual level.

According to Ladd and colleagues (e.g., Ladd et al., 2006), the adjustment of students is concerned with how they successfully adapt to the school environment, which mainly consisted of academic demands and changing social ecologies, and their affects towards school. We measured social competence, distinguished studentship, academic performance, and loneliness as indicators of school and psychological adjustment. We expected that aggression would be negatively associated with later social competence, academic achievement, and distinguished studentship and positively associated with later loneliness.

Furthermore, based on the previous discussion, we hypothesized that classroom cultural norms would have significant moderating effects on the individual-level relations between aggression and later adjustment. The moderating effects might be consistent with two models. Specifically, according to the stress-buffering model (Cohen & Wills, 1985; Rutter, 2013), we expected that aggression would be negatively associated with later social competence, academic achievement, and distinguished studentship and positively associated with later loneliness more evidently in classrooms with a higher group-oriented norm or a lower self-oriented norm. According to the resource-potentiating model (Kupersmidt et al., 1995), we expected that aggression would be positively associated with later social competence, academic achievement, and distinguished studentship and negatively associated with later loneliness more evidently in classrooms with a higher self-oriented norm or lower group-oriented norm.

## Methods

### Participants

This study included 2,671 junior high school students (1,274 boys, 47.7%), initially in seventh grade, in a region in east China. The average age of the students was 12.91 years (*SD* = 6.69 months). The region consisted of rapidly developing towns, small cities, and surrounding areas, which represented a context with a mixture of diverse cultural values (Zhao et al., 2023). The students were from 58 classes in 8 public schools, which were randomly selected from the region, with an average class size of 46 students. The public schools serve students within the residential areas. Under the Chinese domestic curriculum, the structure and organization of the public schools are similar. Students share the same class schedules with peers in the class, and different classes also have highly similar schedules and courses. Students typically stay in the same class with the same classmates over the years and are not allowed to switch classes. A head teacher is responsible for the social and daily activities of the class and usually teaches one of the major subjects. From the original sample, 2,465 students or 92.3% (1,164 boys, 47.2%) participated in a follow up study one year later.

Of the students, 52.9% of them were the only child of the family and the others had one or more siblings. In the sample, 87.7% of the fathers and 94.2% of the mothers had an education of junior high school or lower, 10.2% of the fathers and 5.0% of the mothers had an education of senior high school, and 2.1% of the fathers and 0.8% of the mothers had an education of above high school. The family demographic variables had no significant effects on the relations in the study.

### Procedure

Participants were recruited through the schools. All students were invited to participate in the study with no criteria for exclusion. Participants were informed that participation in the study was completely voluntary and there were no consequences if they refused to participate or dropped out during the study. Active written consent was obtained from parents, and active written assent was obtained from the participating students before the study. There were no students in the schools who could not read, comprehend, or write at the level required for the assent form due to visual impairment, learning disability, or other issues. The participation rate was approximately 95% at each time. The study was approved by the institutional review board at Shanghai Normal University. The original data (Time 1) were collected in 2012 to 2013 and the follow up data (Time 2) were collected in 2013 to 2014.

Following the procedures of the original measure, we group administered to the students a sociometric measure of peer preference, peer assessment measures of aggression and social competence. Self-report data of self-orientation, group-orientation, and loneliness were also collected from students through questionnaires. Teachers were asked to complete a rating scale for each student in the classroom concerning his or her learning problems. The grades on Chinese, mathematics, and English and distinguished studentship were obtained from school records. All measures were administered in Mandarin. The members of our research team carefully examined the items in the measures, using a variety of strategies (e.g., repeated discussions in the research group, interviews with students and teachers, and psychometric analysis). The administration of the measures was carried out by a group of psychology faculty and graduate students in China. Extensive explanations of the procedure were provided during administration. No evidence was found that participants had difficulties understanding the procedure or the items in the measures. The measures of cultural values, aggression, and peer preference were administered at Time 1 and the measures of adjustment were administered in both Times 1 and 2.

### Measures

#### Self-and Group-Orientations

Self- and group orientations were assessed with the Children’s Cultural Values Scale (Chen et al., 2012b). There were 10 items assessing self-orientation (e.g., “I like to be unique and different from others in many aspects,” “I rely on myself, not others.”) and 10 items assessing group-orientation (e.g., “The groups’ decision should be respected by every group member,” “It’s important to maintain harmony within the group.”). Participants were asked to rate each item on a scale ranging from 1 to 5 (1 = *not all at true*; 5 = *almost always true*). The average score of the responses was calculated, with higher scores indicating greater self- or group-orientation. The measure has proven to be internally consistent with alphas ranging from 0.64 to 0.82 and correlated with socioemotional functioning in samples of Chinese adolescents (Chen et al., 2012b; Liu et al., 2018). In the current study, the internal reliability for self- and group-orientations was 0.72 and 0.78 at Times 1 and 2, respectively.

#### Peer Preference

Students were asked to nominate up to three classmates with whom they most liked to be and three classmates with whom they least liked to be (positive and negative nominations). Positive and negative nominations received from peers indicated peer acceptance and rejection. As suggested by previous researchers (Coie et al., 1995), both same-sex and opposite-sex nominations were allowed. The nomination scores were standardized within the classroom to account for the differences in the number of nominators. As suggested by other researchers (e.g., Coie et al., 1982), peer preference was calculated by subtracting negative nomination scores from positive nomination scores. This procedure was used and shown to be reliable and valid in Chinese students (e.g., Liu et al., 2017; Zhao et al., 2022).

#### Classroom Cultural Norms

As suggested by other researchers (e.g., Cialdini et al., 1991; Garandeau et al., 2022; Hu et al., 2023), we measured the classroom cultural norms using the within classroom correlation between cultural orientations and peer preference. In the current study, the classroom self-oriented norm ranged from −0.250 to 0.389 with a mean of 0.005 and a standard deviation of 0.128. The classroom group-oriented norm ranged from −0.354 to 0.456 with a mean of 0.041 and a standard deviation of 0.170. The results showed that there were considerable group variations on both classroom self-oriented norm and group-oriented norm.

#### Aggression

Aggression was measured through a peer nomination measure adapted from the *Revised Class Play* (Chen et al., 2005; Masten et al., 1985). Following the procedure outlined by Masten et al. (1985), students were asked to nominate up to three classmates who could best play the role if they were to direct a class play. Both same-sex and opposite-sex nominations were allowed. The measure consisted of seven items (e.g., “Someone who gets into a lot of fights,” “Picks on other kids”). The nomination scores were standardized within the classroom to account for the differences in the number of nominators. The measure has proven to be internally consistent with alphas ranging from 0.86 to 0.93 and correlated with peer rejection and other social problems among Chinese adolescents (e.g., Chen et al., 2012a, 2012b; Zhao et al., 2022). In the current study, the internal reliability was 0.84 and 0.87 at Times 1 and 2, respectively.

#### Social Competence

Participants’ social competence was measured through a peer nomination measure adapted from the *Revised Class Play* (Chen et al., 2005; Masten et al., 1985). The measure of social competence included four items (e.g., “Someone who is a good leader,” “Likes to express his/her own ideas,” “Everyone listens to him/her”). Students were asked to nominate up to three classmates for each of the items that best describe them, and both same-sex and opposite-sex nominations were allowed. The nomination scores were standardized within the classroom to account for the differences in the number of nominators. The measure has been shown to be internally consistent with alphas ranging from 0.85 to 0.90 and correlated with social status in Chinese samples in previous studies (e.g., Gao et al., 2021). The internal reliability was 0.82 and 0.84 at Times 1 and 2, respectively.

#### Academic Performance

Information concerning students’ academic achievement in Chinese, mathematics, and English was obtained from school records. The grades on these three subjects were based on examinations conducted by the school. Following procedures in previous studies (e.g., Liu et al., 2017; Zhao et al., 2022), the grades of Chinese, mathematics, and English were summed and standardized within the class. In addition, the head teacher in each class was asked to rate each of the students in his/her class on learning problems. There were six items assessing students’ learning problems (e.g., “Having difficulties in learning academic subjects,” “Poorly motivate to achieve”). Teachers rated each item on a 5-point scale in terms of how well each item described the students, with higher scores suggesting the student having more teacher-rated learning problems. Consistent with previous studies, (e.g., Zhao et al., 2022), the teacher-rated scores were averaged and standardized within the classroom. This teacher-rated measure has been used and shown to be internally consistent with alphas ranging from 0.79 to 0.83 and correlated with academic achievement and social status in Chinese adolescents (e.g., Chen et al., 2019). The internal reliability for teacher-rated learning problems were 0.79 and 0.80 at Times 1 and 2, respectively, in the present study. Teacher-rated learning problems were significantly correlated with academic grades, *r* = −0.49 and −0.61, *p* < 0.001, in this study. Consistent with previous studies (e.g., Chen et al., 1997), the standardized scores of students’ academic grades and the reverse-coded standardized scores of teacher-rated learning problems were aggregated to form a single index of academic performance.

#### Distinguished Studentship

In Chinese schools, students who are considered to be socially and intellectually competent and outstanding may be nominated by classmates and teachers for and, once approved, receive school or municipal awards for “distinguished students”. There are various levels of “distinguished student” awards, from the class level to the school level, the district level, and the municipal level. Distinguished studentship was coded as follows: students who did not receive any awards received a score of 0; students who received an award in the class level received a score of 1; students who received an award at the school level received a score of 2; students who received an award at the district level received a score of 3; and students who received an award at the municipal level received a score of 4. The scores of distinguished studentship were standardized within the class. Distinguished studentship has been shown to be a valid measure of school competence in Chinese students (e.g., Chen et al., 1995).

#### Loneliness

A 16-item self-report measure, adapted from Asher et al. (1984), was used to measure loneliness. Students were asked to respond to self-statements describing loneliness (e.g., “I feel lonely”, “I feel like others don’t want to play with me”) using a 5-point scale, ranging from 1 = *not at all true* to 5 = *always true*. The average score of the items was calculated, with higher scores indicating greater loneliness. The measure has been shown to be internally consistent with alphas ranging from 0.78 to 0.92 and correlated with depression among Chinese adolescents in previous studies (Chen et al., 2023; Liu et al., 2015, 2017). In this study, the internal reliability was 0.90 and 0.90 at Times 1 and 2, respectively.

### Planned Analyses

Scores of classroom norms for cultural orientations were calculated by the Pearson correlation coefficient between the scores of self- or group-orientation and peer preference within the classrooms (e.g., Hu et al., 2023), with higher scores indicating that self- or group-orientation was more preferred in the classroom. The analysis was conducted in *Mplus* version 8.10. Two-level hierarchical linear modeling (Raudenbush & Bryk, 2002) was conducted to examine the main and moderating effects of class self- and group-oriented norms and aggression for each adjustment variable. Time 1 individual adjustment, individual aggression, individual self-orientation, and individual group-orientation were included as Level 1 predictors, and child gender was included as a control variable at Level 1. Preliminary analyses showed that the proportion of boys and class size did not have significant effects on the relations, and the results with and without controlling them were similar. Therefore, the two variables were not included in the final analysis. Classroom self-oriented norm and classroom group-oriented norm were included as Level 2 predictors. As suggested by Hofmann and Gavin (1998), individual aggression, individual self-orientation, and individual group-orientation were group-mean centered, and classroom self- and group-orientations were grand-mean centered. The two-level full model used in the final analysis is as follows.

- Level 1:
  $${\text{Y}}_{0\text{j}} = {\upbeta}_{0\text{j}} + {\upbeta}_{1\text{j}}\ (\text{Time}1\text{ individual adjustmen}{\text{t}}_{\text{ij}}) + {\upbeta }_{2\text{j}}\ (\text{Individual aggressio}{\text{n}}_{\text{ij}}) + {\upbeta}_{3\text{j}}\ (\text{Individual self}-{\text{orientation}}_{\text{ij}}) + {\upbeta}_{4\text{j}}\ (\text{Individual group}-{\text{orientation}}_{\text{ij}}) + {\upbeta}_{5\text{j}}\ (\text{Gender}) + {\text{r}}_{\text{ij},}$$

- Level 2:
  $$\begin{array}{c}{\upbeta}_{0\text{j}} = {\upgamma}_{00} + {\upgamma}_{01} (\text{Classroom self}-\text{oriented nor}{\text{m}}_{\text{j}}) + {\upgamma}_{02} (\text{Classroom group}-\text{oriented nor}{\text{m}}_{\text{j}}) + {\upmu }_{0\text{j},}\\ {\upbeta}_{1\text{j}} = {\upgamma}_{10} + {\upgamma}_{11} (\text{Classroom self}-\text{oriented nor}{\text{m}}_{\text{j}}) + {\upgamma}_{12} (\text{Classroom group}-\text{oriented nor}{\text{m}}_{\text{j}}) + {\upmu }_{1\text{j}}\end{array}$$

In the Level 1 equation, Y_0j_ indicates Time 2 individual adjustment outcome and β_0j_ is Level 1 intercept, and β_1j_ to β_5j_ are Level 1 slopes for the relations of Time 1 individual adjustment, individual aggression, individual self-orientation, individual group-orientation, and gender with Time 2 individual adjustment. In Level 2 equation, γ_00_ is Level 2 intercept and γ_01_ and γ_02_ are Level 2 slopes for predicting β_0j_; γ_10_ is Level 2 intercept and γ_11_, γ_12_ are Level 2 slopes for predicting β_1j_. In addition, r_ij_ is the Level 1 residual and µ_0j_, µ_1j_ are Level 2 residuals.

## Results

### Descriptive Data

Little’s MCAR test (Little, 1988) indicated that the data were missing completely at random, with χ^2^(360) = 393.18, *p* > 0.05. As suggested by previous researchers, full information maximum likelihood (FIML) estimation was used to handle the missing data (ranging from 7 to 26% for the variables) for children who had incomplete data on the variables (Graham, 2009). At both times, there were significant gender differences in distinguished studentship (*B* = 0.13 and 0.20, *SE* = 0.05 and 0.02, *t* = 2.79 and 4.47, *p*s < 0.01) and academic performance (*B* = 0.51 and 0.40, *SE* = 0.06 and 0.08, *t* = 8.08 and 5.01, *p*s < 0.001), suggesting that girls have higher academic performance and distinguished studentships than boys at both times. At Time 1, boys had lower loneliness (*B* = 0.06, *SE* = 0.03, *t* = 2.15, *p* < 0.05) and higher aggression (*B* = .−0.37, *SE* = 0.03, *t* = −14.02, *p* < 0.001) than girls. The means and standard deviations for both boys and girls as well as the correlations among the variables are presented in Table 1 and Table 2.

**Table 1:** Means and standard deviations of the variables

	Boys		Girls	
	Mean	SD	Mean	SD
*Time 1*				
Self-orientation	3.13	.61	3.07	.59
Group-orientation	3.52	.66	3.54	.60
Aggression	.18	.87	-.19	.41
Peer preference	-.10	1.62	.12	1.48
Social competence	-.03	.73	.02	.86
Academic performance	-.26	1.64	.25	1.46
Distinguished Studentship	-.05	.92	.08	1.06
Loneliness	2.03	.66	2.08	.66
*Time 2*				
Social competence	-.01	.75	.03	.88
Academic performance	-.19	1.74	.22	1.51
Distinguished studentship	-.08	.86	.12	1.16
Loneliness	2.11	.61	2.16	.61

**Table 2:** Correlations among variables

	1	2	3	4	5	6	7	8	9	10	11
*Time 1*											
1. Self-orientation											
2. Group-orientation	.42***										
3. Peer preference	.00	.07***									
4. Aggression	.14***	-.01	-.36***								
5. Social competence	.24***	.11*	.16***	.17***							
6. Academic performance	.18**	.11***	.30***	-.10***	.41***						
7. Distinguished studentship	.13***	.10***	.15***	-.03	.43***	.37***					
8. Loneliness	-.24***	-.39***	-.19***	-.02	-.11***	-.13***	.07***				
*Time 2*											
9. Social competence	.23***	.11***	.16***	.13***	.85***	.40***	.40***	-.11***			
10. Academic performance	.22***	.15***	.30***	-.07***	.42***	.78***	.33***	-.12***	.43***		
11. Distinguished studentship	.15***	.11***	.14***	-.01	.45***	.35***	.39***	-.09***	.45***	.43***	
12. Loneliness	-.18***	-.29***	-.14***	-.04***	-.10***	-.10***	-.07***	.54***	-.11***	-.08***	-.09***

^*^p < .05, **p < .01, *** *p* < .001

### Effects of Classroom Cultural Norms

The results regarding the main effects of Level 1 and Level 2 variables and the cross-level interactions between self-oriented and group-oriented class norms and individual aggression are presented in Table 3. For within-class associations, self-orientation positively predicted T2 social competence and distinguished studentship and negatively predicted T2 loneliness. Group-orientation positively predicted T2 academic performance and negatively predicted T2 loneliness. Classroom cultural norms did not have significant main effects on T2 adjustment variables, suggesting that students in classrooms with different self- and group-oriented norms, in general, tended to have similar T2 adjustments.

**Table 3:** Effects of time 1 predictors on time 2 adjustment outcome variables

T2 Outcome				
	*β*	*SE*	*t* value	95% CI
*Social competence*				
Gender	-.01	.02	-.60	[-.05, .03]
Social competence	.85	.02	48.45***	[.82, .89]
Aggression	-.00	.01	−1.14	[-.03, .03]
Self-orientation	.04	.02	1.99*	[.00, .07]
Group-orientation	.00	.02	.29	[-.03, .03]
Classroom self-oriented norm	.03	.05	.55	[-.06, .12]
Classroom group-oriented norm	.00	.03	.01	[-.06, .06]
Aggression*Classroom self-oriented norm	.17	.15	1.14	[-.12, .45]
Aggression*Classroom group-oriented norm	-.21	.10	−2.05*	[-.41, -.01]
*Academic Performance*				
Gender	.04	.07	.47	[-.11, .18]
Academic performance	.80	.04	22.78***	[.73, .87]
Aggression	-.02	.04	-.38	[-.10, .07]
Self-orientation	.06	.04	1.78	[-.01, .13]
Group-orientation	.09	.04	2.11*	[.01, .17]
Classroom self-oriented norm	-.24	.27	-.91	[-.77, .28]
Classroom group-oriented norm	-.04	.13	-.27	[-.29, .22]
Aggression*Classroom self-oriented norm	.27	.35	.79	[-.41, .95]
Aggression*Classroom group-oriented norm	-.46	.24	−1.97*	[-.93, -.00]
*Distinguished Studentship*				
Gender	.15	.04	3.45**	[.07, .24]
Distinguished studentship	.38	.04	8.67***	[.29, .46]
Aggression	.05	.04	1.50	[-.02, .12]
Self-orientation	.20	.05	3.82***	[.10, .31]
Group-orientation	.07	.04	1.65	[-.01, .16]
Classroom self-oriented norm	-.07	.09	-.84	[-.25, .10]
Classroom group-oriented norm	.08	.07	1.15	[-.06, .21]
Aggression*Classroom self-oriented norm	.39	.31	1.25	[-.22, 1.01]
Aggression*Classroom group-oriented norm	-.56	.18	−3.03**	[-.92, -.20]
*Loneliness*				
Gender	.01	.02	.36	[-.03, .05]
Loneliness	.47	.02	19.54***	[.42, .52]
Aggression	-.06	.02	−2.28*	[-.10, -.02]
Self-orientation	-.04	.02	−2.03*	[-.09, -.00]
Group-orientation	-.06	.03	−2.38*	[-.11, -.01]
Classroom Self-oriented norm	.01	.18	.04	[-.34, .35]
Classroom Group-oriented norm	-.07	.12	-.59	[-.32, .17]
Aggression*Classroom self-oriented norm	-.29	.18	−1.63	[-.64, .06]
Aggression*Classroom group-oriented norm	.37	.18	2.06*	[.02, .72]

^*^p < .05, **p < .01, ***p < .001

**Fig. 1:**
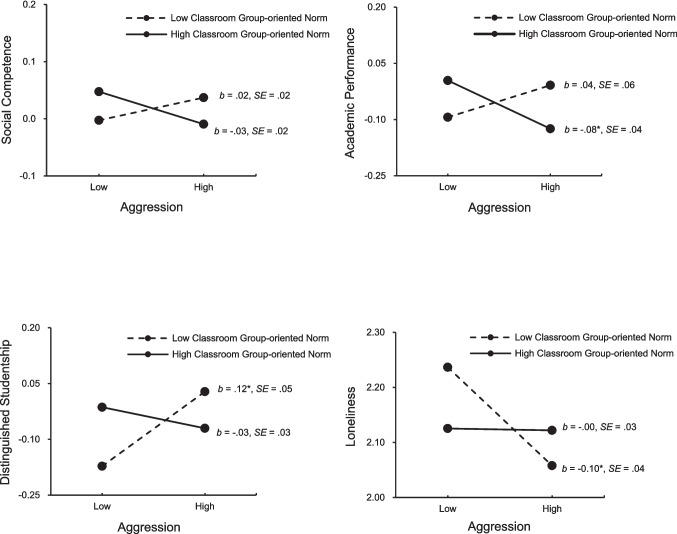
Moderating effects of classroom group-oriented norm on the relations between time 1 aggression and time 2 adjustment outcomes (*p < .05)

The results showed significant interactions between Time 1 Aggression × Classroom group-oriented norm in predicting T2 social competence, academic performance, distinguished studentship, and loneliness. In order to better understand the nature of the interactions, we examined simple slopes of the regression of each of the T2 variables on T1 aggression at a high value and a low value (1 standard deviation above and 1 standard deviation below the mean) of classroom group-oriented norm, as described by Aiken and West (1991). The results are presented in Fig. 1.

As illustrated in the figure, simple slopes between Time 1 aggression and social competence were not significant. The relation tended to be marginally significant and negative in classes with high classroom group-oriented norm (*p* = 0.07) but not significant in classes with low classroom group-oriented norm (*p* = 0.30). Time 1 aggression was negatively associated with Time 2 academic performance in classrooms with high group-oriented norm; the association was not significant in classes with low classroom group-oriented norm. Time 1 aggression was also positively associated with Time 2 distinguished studentship and negatively associated with loneliness in classrooms with low classroom group-oriented norm, but the associations were not significant in classrooms with high classroom group-oriented norm.

**Fig. 2:**
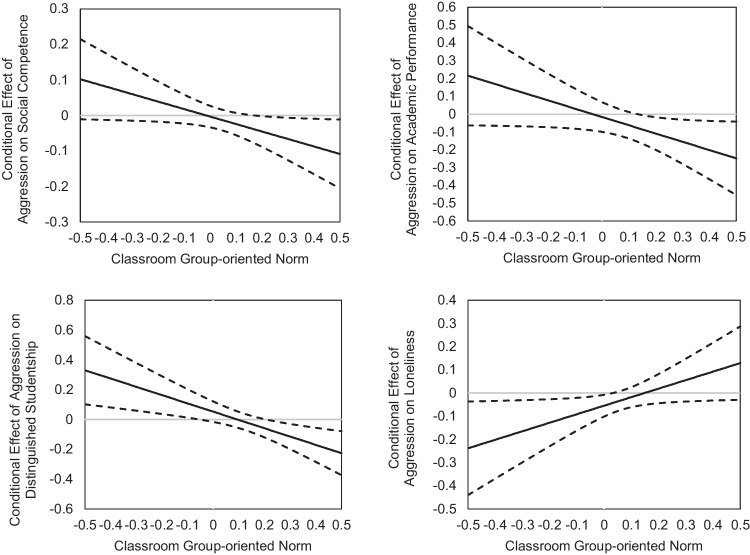
Johnson-neyman plot for the conditional effect of time 1 aggression in predicting time 2 adjustment outcomes

Johnson-Neyman technique was used to plot the conditional effect of aggression in predicting later outcomes. As shown in Fig. 2, the effects of aggression were significant when the classroom group-oriented norm was higher than 0.17 and 0.13 for social competence and academic performance. The effects of aggression were significant when group-oriented norms were lower than −0.05 and higher than 0.20 for distinguished studentship. The effect of aggression was significant when the classroom group-oriented norm was lower than 0.03 for loneliness.

## Discussion

Previous studies have indicated the role of the classroom norms in shaping adolescents’ development (e.g., Garandeau et al., 2022; Hu et al., 2023). The significance of classroom norms may be particularly evident in Chinese adolescents because students typically stay in the same classroom and interact with the same peers throughout middle school. The collectivistic context and associated school practices (e.g., the public evaluation process in which students are regularly evaluated by peers) may further facilitate the regulatory function of classroom norms in individual development. The primary purpose of the current study was to investigate the moderating effects of classroom cultural norms on the relations between aggression and school adjustment among Chinese adolescents. The results indicated that classroom group-oriented norm significantly moderated the relations between aggression and later adjustment variables. These results suggest that classroom cultural norms, particularly group-oriented norm, may be a crucial factor in adolescents’ development in Chinese schools.

### The Moderating Effects of Classroom Cultural Norms on the Relations Between Aggression and Adjustment

Aggressive behavior is discouraged in Chinese schools because it poses a significant threat to group harmony and interpersonal relationships (Chen, 2023; Chen & French, 2008). Previous studies with Chinese students showed that aggression in early adolescents was associated with social-behavioral and academic problems (e.g., Chen et al., 2012a, 2012b; Liu et al., 2012). The results of the present study indicated that how aggression was associated with later adjustment outcomes largely depended on the classroom cultural norms.

The results first showed that group-oriented classroom norm moderated the relations of individual aggression with academic and social competence. Consistent with our hypothesis, aggression significantly and negatively predicted later academic performance and marginally and negatively predicted social competence in classrooms where group-orientation was more endorsed. In classrooms with a high group-oriented norm, adolescents with high aggression are likely to acquire unfavorable experiences with others. In this environment, adolescents with higher aggression may receive more negative evaluations and responses from peers, which may make them to be less confident during social interactions and obtain fewer opportunities to practice social skills and acquire leadership status. At the same time, they are less likely to receive help and support from their peers and teachers for academic work, which may undermine their academic performance. Thus, the highly group-oriented classroom norm served to exacerbate the problems that adolescents with high aggression display. Compared to their counterparts in classrooms with a high group-orientation, these adolescents in classrooms where group-orientation was not valued did not develop evident social and academic problems. A low level of group-orientation in the classroom appeared to be a protective factor buffering against maladaptive social and academic development of adolescents with high aggression (Cohen & Wills, 1985; Rutter, 2013).

Group-oriented classroom norm also moderated the relation between aggression and later distinguished studentships and loneliness. More specifically, aggression positively predicted distinguished studentship and negatively predicted loneliness in classrooms with a low group-oriented norm but not in classrooms with a high group-oriented norm. The low group-oriented norm appeared to enhance the beneficial effect of aggression on later distinguished studentship and loneliness, suggesting that adolescents higher on aggression were more likely than others to gain social recognition and less likely to experience loneliness in this environment. In these classrooms, aggression might not be perceived as problematic and deviant as in classrooms with a high group-oriented norm. Adolescents high on aggression might be regarded as socially assertive and autonomous. Thus, they might not receive the same level of social disproval as their counterparts did in classrooms with a high group-oriented norm. The relatively supportive environment allowed them to engage in more social activities, which increased their positive social experience and psychological well-being. These results supported the resource-potentiating model (Kupersmidt et al., 1995) – adolescents’ aggressive behavior was a useful strategy that was “potentiated” by the low group-oriented classroom norm for students to obtain social and psychological benefits (Hawley, 2007; Hawley & Bower, 2018).

The results indicated that consistent with previous findings (e.g., Liu et al., 2018), self-orientation positively contributed to later social competence, academic achievement, and distinguished students and negatively contributed to later loneliness after the stability was controlled at the individual level. The results suggest that in contemporary China, as individual independence and other self-oriented qualities may be perceived as desirable (Chen, 2023), adolescents who behave in a self-directed manner may be viewed as competent and likely receive support and assistance in school performance. Moreover, self-orientation and associated attitudes such as self-confidence may help adolescents reduce psychological difficulties, such as the feelings of loneliness (Liu et al., 2018). Unlike classroom group-oriented norms, however, self-oriented norms in the classroom did not have significant effects in moderating the relations between aggression and adjustment. The results suggest that the group-oriented norm may reflect the classroom climate with more salient significance (e.g., the belief about classroom solidarity) than the self-oriented norm. As a result, whereas individual self-orientation predicted social and psychological adjustment, the classroom self-oriented norm did not have evident effects on adolescents’ adjustment.

In sum, the results of the current study indicated the moderating effects of classroom group-oriented norm on the relations between aggression and adjustments in adolescents. In classrooms where group-oriented values were endorsed, aggressive behavior negatively contributed to later competence and academic performance. In contrast, in classrooms where group-oriented values were less preferred, aggression positively predicted later distinguished studentship and negatively predicted later loneliness. The results of the study underscore the importance of the role of classroom contexts in shaping the adjustment outcomes of aggressive behavior.

### Limitations and Future Directions

Several limitations and weaknesses in the study should be noted. First, though the current study used a longitudinal design, it is correlational in nature. One should be cautious in interpreting the results in terms of the causality. Second, this longitudinal study only included two waves of data. The effects of aggression on adjustment are likely to be a continuous and long-term process (Chen et al., 2012a, 2012b). According to the developmental cascade perspective (e.g., Masten & Cicchetti, 2010), behavioral characteristics including aggression are likely to exert influence on individual development gradually and progressively over time. Thus, multiwave longitudinal data should be collected in future research to better understand the relation among aggression, classroom cultural norms, and adjustment outcomes. Third, the data were collected mostly from peer-assessments, teacher ratings, and adolescents’ self-reports in this study. The measures have been commonly used and shown to be reliable and valid in previous studies with school age children and adolescents in China and other countries (e.g., Chen et al., 2005, 2012a, 2012b; Liu et al., 2015, 2017; Masten et al., 1985; Rubin et al., 2015). Nevertheless, given the potential biases in self-report measures, it will be important to collect data on adolescents’ behaviors (e.g., aggressive behavior) and adjustment from other sources, such as observations, in future research. Fourth, the present study focused on classroom cultural norms as indexed by the levels of endorsement of group-oriented and self-oriented values by students in the classroom. It will be interesting to investigate the role of teachers’ preference for self- and group-oriented values in relations between aggression and adjustment in students. Finally, the current study was conducted in a region consisting of towns, small cities, and surrounding areas in east China. There are substantial differences across regions in China, and one should be cautious in generalizing the results to other regions.

Despite the limitations, the current study provided valuable information about how classroom cultural norms play a role in moderating the relations between aggression and school and psychosocial adjustments among Chinese adolescents. The study also has important practical implications. Teachers should be aware of the classroom environment, especially the classroom cultural norms. In classrooms where group orientation is highly preferred, for example, teachers should pay particular attention to problems that aggressive students may experience and design strategies to help them improve their social and academic performance.

## Data Availability

The data and material for the current study are not publicly available but are available from the corresponding author on reasonable request.
